# Supplementary material for: Infant vocal category exploration as a foundation for speech development
Source: PLoS One. 2024 May 29;19(5):e0299140. doi: 10.1371/journal.pone.0299140 (PMC11135693; doi:10.1371/journal.pone.0299140)
Supplement: S1 File — (ZIP) [file pone.0299140.s001.zip › IRB/2143_IRB appletter 2019-present.pdf]

Institutional Review Board
Division of Research and Innovation
Office of Research Compliance
University of Memphis
315 Admin Bldg
Memphis, TN 38152-3370

PI: David Oller
Co-Investigator:
Advisor and/or Co-PI:
Department:School of Communication Sciences and Disorders (CSD)
Study Title:Vocal and Speech Development
IRB ID: 2143
Submission Type: Renewal

Date:Jun 12, 2019 9:14 AM CDT

Dear David Oller,

The U.S. Department of Health and Human Services and Office for Human Subjects Protections announced that the revisions to the Common Rule went into effect January 19, 2019. Under the new regulations protocols in the expedited category no longer require continuing review. As investigators you are responsible for complying with the following:

1. When the project is finished a completion submission is required.
2. Any changes to the approved protocol requires board approval prior to implementation.
3. When necessary submit incidents/adverse events to the board for review
4. Human subjects training is required to be kept current at citiprograms.org every 2 years

For any additional question or concerns please contact us at irb@memphis.edu or 901.678.2705

Thank you,

James Whelan, Ph.D.
Institutional Review Board Chair
The University of Memphis
