# Supplementary material for: Infant vocal category exploration as a foundation for speech development
Source: PLoS One. 2024 May 29;19(5):e0299140. doi: 10.1371/journal.pone.0299140 (PMC11135693; doi:10.1371/journal.pone.0299140)
Supplement: S1 File — (ZIP) [file pone.0299140.s001.zip › IRB/2143Approval Letter_2018-19.pdf]

Institutional Review Board
Office of Sponsored Programs
University of Memphis
315 Admin Bldg
Memphis, TN 38152-3370

PI: David Oller
Co-Investigator:
Advisor and/or Co-PI:
Department: School of Comm Sci & Disorders
Study Title: Vocal and Speech Development
IRB ID: 2143
Submission Type: Renewal
Level of Review: Expedited

IRB Meeting Date:
Decision: Approved
Approval Date: July 25, 2018
Expiration Date: July 25, 2019

Research Notes:
Findings:

The IRB has reviewed the renewal request.

Approval of this project is given with the following obligations:
1. If this IRB approval has an expiration date, an approved renewal must be in effect to continue the project prior to that date. If approval is not obtained, the human consent form(s) and recruiting material(s) are no longer valid and any research activities involving human subjects must stop.
2. When the project is finished or terminated, a completion form must be completed and sent to the board.
3. No change may be made in the approved protocol without prior board approval, whether the approved protocol was reviewed at the Exempt, Expedited or Full Board level.
4. Exempt approval are considered to have no expiration date and no further review is necessary unless the protocol needs modification.

Thank you,
James P. Whelan, Ph.D.

Institutional Review Board Chair
The University of Memphis.
Note: Review outcomes will be communicated to the email address on file. This email should be considered an official communication from the UM IRB.
