# Supplementary material for: Infant vocal category exploration as a foundation for speech development
Source: PLoS One. 2024 May 29;19(5):e0299140. doi: 10.1371/journal.pone.0299140 (PMC11135693; doi:10.1371/journal.pone.0299140)
Supplement: S1 File — (ZIP) [file pone.0299140.s001.zip › IRB/IRB approval #2143 2015-16.pdf]

From: Christopher Wayne Whitehead (cwhitehd) **On Behalf Of** Institutional Review Board
Sent: Friday, July 31, 2015 12:26 PM
To: David Kimbrough Oller (koller)
Subject: IRB Approval 2143

Hello,

The University of Memphis Institutional Review Board, FWA00006815, has reviewed and approved your submission in accordance with all applicable statuses and regulations as well as ethical principles.

PI NAME: David Oller
CO-PI: Eugene Buder
PROJECT TITLE: Vocal and Speech Development
FACULTY ADVISOR NAME (if applicable):
IRB ID: #2143
APPROVAL DATE: 07/31/2015
EXPIRATION DATE: 07/31/2016
LEVEL OF REVIEW: Expedited

Please Note: Modifications do not extend the expiration of the original approval

Approval of this project is given with the following obligations:

1. If this IRB approval has an expiration date, an approved renewal must be in effect to continue the project prior to that date. If approval is not obtained, the human consent form(s) and recruiting material(s) are no longer valid and any research activities involving human subjects must stop.

2. When the project is finished or terminated, a completion form must be completed and sent to the board.

3. No change may be made in the approved protocol without prior board approval, whether the approved protocol was reviewed at the Exempt, Exedited or Full Board level.

4. Exempt approval are considered to have no expiration date and no further review is necessary unless the protocol needs modification.

Approval of this project is given with the following special obligations:

Thank you,

James P. Whelan, Ph.D.

Institutional Review Board Chair

The University of Memphis.

Note: Review outcomes will be communicated to the email address on file. This email should be considered an official communication from the UM IRB. Consent Forms are no longer being stamped as well. Please contact the IRB at IRB@memphis.edu if a letter on IRB letterhead is required.
