# Supplementary material for: Infant vocal category exploration as a foundation for speech development
Source: PLoS One. 2024 May 29;19(5):e0299140. doi: 10.1371/journal.pone.0299140 (PMC11135693; doi:10.1371/journal.pone.0299140)
Supplement: S1 File — (ZIP) [file pone.0299140.s001.zip › IRB/IRB0000097674.pdf]

TO: Gordon Ramsay, MD
Principal Investigator
SOM: Peds: Marcus Center

DATE: December 19, 2017

RE: **Expedited Approval**
IRB00097674
Pathways of Social Contingency for Navigating Developmental Landscapes of Risk in ASD: Developmental Progressions and Pivotal Transitions in Infant-Caregiver Vocal Interaction

Thank you for submitting a new application for this protocol. This research is eligible for expedited review under 45 CFR.46.110 and/or 21 CFR 56.110 because it poses minimal risk and fits the regulatory categories F[5], F[6], and F[7] as set forth in the Federal Register. The Emory IRB reviewed it by expedited process on **12/18/2017** and granted approval effective from **12/18/2017** through **12/17/2018**. Thereafter, continuation of human subjects research activities requires the submission of a renewal application, which must be reviewed and approved by the IRB prior to the expiration date noted above. Please note carefully the following items with respect to this approval:

- Scientific Protocol
- Family Information and Demographics Form
- Family Medical History Form
- Facebook Advertisement
- Facebook Post
- Recruitment Direct Mailing
- Recruitment Direct Email
- Recruitment Flyer
- Recruitment Rack Card
- Combined Consent/Permission Form
- Contact Information Form
- Payment Card Information Form
- Recording Permission Form (Adult/Child)
- Release of Healthcare Information Form (Adult)
- Release of Healthcare Information Form (Child)
- Revocation Letter
- The IRB verified that grant application # MH100029 corresponds to this research protocol. The title of the grant is "Cycles of Social Contingency in Autism: Pivotal Transitions that Shape Infant Brain-Behavior Development in Human & Model Systems".
- This study meets the criteria for permissible clinical research with children as set forth at 45 CFR 46.404/21 CFR 50.51. One/two parent or guardians’ signed permission is sufficient to enroll minor subjects.

Parent or guardian permission must be obtained, as well as assent of the child, as per 45 CFR 46.408/21 CFR 50.55 and Emory's age-based assent guidelines, which are outlined in your approved assent form.

- A partial waiver of HIPAA authorization has been approved by the IRB for the purpose of identifying potential subjects for this protocol. As subjects are contacted, you are required to obtain their HIPAA authorization.

Any reportable events (e.g., unanticipated problems involving risk to subjects or others, noncompliance, breaches of confidentiality, HIPAA violations, protocol deviations) must be reported to the IRB according to our Policies & Procedures at www.irb.emory.edu, immediately, promptly, or periodically. Be sure to check the reporting guidance and contact us if you have questions. Terms and conditions of sponsors, if any, also apply to reporting.

Before implementing any change to this protocol (including but not limited to sample size, informed consent, study design, you must submit an amendment request and secure IRB approval.

In future correspondence about this matter, please refer to the IRB file ID, name of the Principal Investigator, and study title. Thank you

Leslie Garrett
Senior Research Protocol Analyst
This letter has been digitally signed

CC:	Edwards	Morgan	SOM: Peds: Marcus Center
	Fleurissaint	Rose-Milord	SOM: Peds: Marcus Center
	Trumbull	Ashley	SOM: Peds: Marcus Center

Ghai	Shweta	SOM: Peds: Marcus Center
Jones	Warren	SOM: Peds: Marcus Center
Klin	Ami	SOM: Peds: Marcus Center

TO: Gordon Ramsay, MD
Principal Investigator
SOM: Peds: Marcus Center

DATE: December 12, 2018

RE: **Continuing Review Expedited Approval**
CR1_IRB00097674

IRB00097674
Pathways of Social Contingency for Navigating Developmental Landscapes of Risk in ASD: Developmental Progressions and Pivotal Transitions in Infant-Caregiver Vocal Interaction

Dear Dr. Ramsay,

Thank you for submitting a renewal application for this protocol. The Emory IRB reviewed it by the expedited process on **12/12/2018**, per 45 CFR 46.110, the Federal Register expeditable categories F[5],F[6], F[7] and/or 21 CFR 56.110. This reapproval is effective from **12/12/2018** through **12/11/2019**. Thereafter, continuation of human subjects research activities requires the submission of another renewal application, which must be reviewed and approved by the IRB prior to the expiration date noted above. Please note carefully the following items with respect to this reapproval:

- Protocol:
  - Scientific Protocol
  - 97674.Subpart D Worksheet.doc
- Consents:
  - Combined Consent/Permission Form
    Contact Information Form
    Payment Card Information Form
    Recording Permission Form (Adult/Child)
    Release of Healthcare Information Form (Adult)
    Release of Healthcare Information Form (Child)
    Revocation Letter

Any reportable events (e.g., unanticipated problems involving risk to subjects or others, noncompliance, breaches of confidentiality, HIPAA violations, protocol deviations) must be reported to the IRB according to our Policies & Procedures at www.irb.emory.edu, immediately, promptly, or periodically. Be sure to check the reporting guidance and contact us if you have questions. Terms and conditions of sponsors, if any, also apply to reporting.

Before implementing any change to this protocol (including but not limited to sample size, informed consent, and study design), you must submit an amendment request and secure IRB approval.

In future correspondence about this matter, please refer to the IRB file ID, name of the Principal Investigator, and study title. Thank you.

Sincerely,

Reonna Taylor, MPH
IRB Analyst Assistant
This letter has been digitally signed

CC:			
	Edwards	Morgan	SOM: Peds: Marcus Center
	Fleurissaint	Rose-Milord	SOM: Peds: Marcus Center
	Trumbull	Ashley	SOM: Peds: Marcus Center

Ghai	Shweta	SOM: Peds: Marcus Center
Jones	Warren	SOM: Peds: Marcus Center
Klin	Ami	SOM: Peds: Marcus Center

Emory University
1599 Clifton Road, 5th Floor - Atlanta, Georgia 30322
Tel: 404.712.0720 - Fax: 404.727.1358 - Email: irb@emory.edu - Web: http://www.irb.emory.edu/
An equal opportunity, affirmative action university

TO: Gordon Ramsay, MD
Principal Investigator
SOM: Peds: Marcus Center

DATE: 11/22/2019

RE: **Continuing Review Expedited Approval**
CR2_IRB00097674

IRB00097674
Pathways of Social Contingency for Navigating Developmental Landscapes of Risk in ASD: Developmental Progressions and Pivotal Transitions in Infant-Caregiver Vocal Interaction

Thank you for submitting a renewal application for this protocol. The Emory IRB reviewed it by the expedited process on , per 45 CFR 46.110, the Federal Register expeditable categories F4, F5, F7, and/or 21 CFR 56.110. This reapproval is effective from
11/22/2019 through **11/21/2020**. Thereafter, continuation of human subjects research activities requires the submission of another renewal application, which must be reviewed and approved by the IRB prior to the expiration date noted above. Please note carefully the following items with respect to this reapproval:

- Combined Consent/Permission Form
- Contact Information Form
- Payment Card Information Form
- Recording Permission Form (Adult/Child)
- Release of Healthcare Information Form (Adult)
- Release of Healthcare Information Form (Child)
- Revocation Letter

Any reportable events (e.g., unanticipated problems involving risk to subjects or others, noncompliance, breaches of confidentiality, HIPAA violations, protocol deviations) must be reported to the IRB according to our Policies & Procedures at www.irb.emory.edu, immediately, promptly, or periodically. Be sure to check the reporting guidance and contact us if you have questions. Terms and conditions of sponsors, if any, also apply to reporting.

Before implementing any change to this protocol (including but not limited to sample size, informed consent, and study design), you must submit an amendment request and secure IRB approval.

In future correspondence about this matter, please refer to the IRB file ID, name of the Principal Investigator, and study title. Thank you.

Sincerely,

Kalifa Alexander, MPH
IRB Analyst Assistant
This letter has been digitally signed

CC:

Antonio	Mikafui	SOM: Peds: Marcus Center
Edwards	Morgan	SOM: Peds: Marcus Center

Ghai	Shweta	SOM: Peds: Marcus Center
Jones	Warren	SOM: Peds: Marcus Center
Klin	Ami	SOM: Peds: Marcus Center

IRB FULL BOARD CONTINUING REVIEW APPROVAL

November 23, 2020

Gordon Ramsay, PhD

gjramsa@emory.edu

Title:	Pathways of Social Contingency for Navigating Developmental Landscapes of Risk in ASD: Developmental Progressions and Pivotal Transitions in Infant-Caregiver Vocal Interaction
Principal Investigator:	Gordon Ramsay, PhD
IRB ID:	CR001-IRB00097674
Funding:	Name: Federal Agency, Emory EPEX ID: 39477, Funding Source ID: NIMH - National Institute of Mental Health
Documents Reviewed:	• Combined Consent/Permission Form, Category: Consent Form;<br>• Contact Information Form, Category: Consent Form;<br>• Demographic Information Form, Category: Surveys, Questionnaires, Interview Guides;<br>• Payment Card Information Form, Category: Consent Form;<br>• Recording Permission Form (Adult/Child), Category: Consent Form;<br>• Release of Healthcare Information Form (Adult), Category: Consent Form;<br>• Release of Healthcare Information Form (Child), Category: Consent Form;<br>• Revocation Letter, Category: Consent Form;<br>• Scientific Protocol, Category: IRB Protocol;

Dear Dr. Ramsay:

The Emory IRB reviewed the above-referenced study at its convened meeting on 11/19/2020 and granted approval effective from 11/19/2020 through 11/18/2021. Thereafter, continuation of human subjects' research activities requires the submission of a renewal application, which must be reviewed and approved by the IRB prior to the expiration date noted above.

Please note carefully the following items with respect to this approval:

• Combined Consent/Permission Form
• Contact Information Form
• Demographic Information Form
• Payment Card Information Form
• Recording Permission Form (Adult/Child)
• Release of Healthcare Information Form (Adult)
• Release of Healthcare Information Form (Child)
• Revocation Letter
• Scientific Protocol

In conducting this protocol, you are required to follow the requirements listed in the Emory Policies and Procedures, which can be found at our IRB website.

Sincerely,

Parul Reddy
Research Protocol Analyst

Your stamped consent form is available under the "Documents" tab.

Now that your submission has been approved, please take a few moments to complete the Emory IRB Satisfaction Survey. We will use your responses to improve our service to the Emory research community. We appreciate your feedback!

IRB CONTINUING REVIEW APPROVAL

November 8, 2021

Gordon Ramsay, PhD

gjramsa@emory.edu

Title:	Pathways of Social Contingency for Navigating Developmental Landscapes of Risk in ASD: Developmental Progressions and Pivotal Transitions in Infant-Caregiver Vocal Interaction
Principal Investigator:	Gordon Ramsay, PhD
IRB ID:	CR002-IRB00097674
Funding:	Name: Federal Agency, Emory EPEX ID: 39477, Funding Source ID: NIMH - National Institute of Mental Health
IND, IDE or HDE:	None
Documents Reviewed:	• Combined Consent/Permission Form, Category: Consent Form;<br>• Contact Information Form, Category: Consent Form;<br>• Recording Permission Form (Adult/Child), Category: Consent Form;<br>• Release of Healthcare Information Form (Adult), Category: Consent Form;<br>• Release of Healthcare Information Form (Child), Category: Consent Form;<br>• Revocation Letter, Category: Consent Form;<br>• Scientific Protocol, Category: IRB Protocol;

Dear Dr. Gordon Ramsay:

Thank you for submitting a renewal application for this protocol. The Emory IRB approved it by the expedited process on 11/4/2021, per 45 CFR 46.110, the Federal Register expeditable categories F[5, 6, 7(b)], and/or 21 CFR 56.110.

This reapproval is effective from 11/4/2021 through 11/3/2022. Thereafter, continuation of human subjects research activities requires the submission of another renewal application, which must be reviewed and approved by the IRB prior to the expiration date noted above.

Please note carefully the following items with respect to this approval:

- Combined Consent/Permission Form, Category: Consent Form;
- Contact Information Form, Category: Consent Form;
- Recording Permission Form (Adult/Child), Category: Consent Form;
- Release of Healthcare Information Form (Adult), Category: Consent Form;
- Release of Healthcare Information Form (Child), Category: Consent Form;
- Revocation Letter, Category: Consent Form;
- Scientific Protocol, Category: IRB Protocol;

In conducting this protocol, you are required to follow the requirements listed in the Emory Policies and Procedures, which can be found at our IRB website.

Sincerely,

Chelsea Cassara
IRB Analyst Assistant

Your stamped consent form is available under the "Documents" tab.

Now that your submission has been approved, please take a few moments to complete the Emory IRB Satisfaction Survey. *We will use your responses to improve our service to the Emory research community. We appreciate your feedback!*

IRB APPROVAL: CONTINUING REVIEW WITH ACCOMPANYING MODIFICATION

October 27, 2022

Gordon Ramsay, PhD

gjramsa@emory.edu

Title:	Pathways of Social Contingency for Navigating Developmental Landscapes of Risk in ASD: Developmental Progressions and Pivotal Transitions in Infant-Caregiver Vocal Interaction
Principal Investigator:	Gordon Ramsay, PhD
IRB ID:	MODCR001-IRB00097674
Funding:	Name: Federal Agency, Emory EPEX ID: 39477, Funding Source ID: NIMH - National Institute of Mental Health
IND, IDE or HDE:	None
Documents Reviewed:	• Combined Consent/Permission Form, Category: Consent Form;<br>• Contact Information Form, Category: Consent Form;<br>• Payment Card Information Form, Category: Consent Form;<br>• Recording Permission Form (Adult/Child), Category: Consent Form;<br>• Release of Healthcare Information Form (Adult), Category: Consent Form;<br>• Release of Healthcare Information Form (Child), Category: Consent Form;<br>• Revocation Letter, Category: Consent Form;<br>• Scientific Protocol, Category: IRB Protocol;

Dear Dr. Gordon Ramsay:

Thank you for submitting an application for continuing review with accompanying modifications for this protocol. The Emory IRB approved this submission by the expedited process on 10/27/2022, per 45 CFR 46.110, the Federal Register expeditable categories F[5, 6, 7(a, b)], and/or 21 CFR 56.110.

The reapproval is effective from 10/27/2022 through 10/26/2023. Thereafter, continuation of human subjects research activities requires the submission of another renewal application, which must be reviewed and approved by the IRB prior to the expiration date noted above. **OR** No further annual IRB review is required, as permitted under the 2018 Common Rule.

The following modifications were included in this review:

- Study Staff Changes

Please note carefully the following items with respect to this reapproval:

- Combined Consent/Permission Form, Category: Consent Form;
- Contact Information Form, Category: Consent Form;
- Payment Card Information Form, Category: Consent Form;
- Recording Permission Form (Adult/Child), Category: Consent Form;
- Release of Healthcare Information Form (Adult), Category: Consent Form;
- Release of Healthcare Information Form (Child), Category: Consent Form;
- Revocation Letter, Category: Consent Form;
- Scientific Protocol, Category: IRB Protocol;

In conducting this protocol, you are required to follow the requirements listed in the Emory Policies and Procedures, which can be found at our IRB website.

Sincerely,

Chelsea Cassara, BS
IRB Analyst Assistant

Your stamped consent form is available under the "Documents" tab.

Now that your submission has been approved, please take a few moments to complete the Emory IRB Satisfaction Survey. We will use your responses to improve our service to the Emory research community. We appreciate your feedback!
