# Supplementary material for: Infant vocal category exploration as a foundation for speech development
Source: PLoS One. 2024 May 29;19(5):e0299140. doi: 10.1371/journal.pone.0299140 (PMC11135693; doi:10.1371/journal.pone.0299140)
Supplement: S3 File — It also includes waveforms (S1-S5 Waveforms) and spectrograms of each vocal category (S1-S10 File) along with relevant details. (PDF) [file pone.0299140.s003.pdf]

Supporting Information

Coding categories, definitions and coding criteria

The coding ignored vegetative sounds such as coughing, sneezing, and burping, none of which are treated as signals evolved as communications. It also ignored effort grunts, which we treat as artifacts of straining or movement. Coding of potentially communicative signals was conducted at the “utterance” level for all categories [1]. An utterance was defined as a breath group, where an inhalation was always taken to constitute an utterance boundary. Utterances could include silences (glottal holds or consonant-like closures, for example) during which no inhalation occurred. Also, a period without an inhalation could be judged, at the coder’s discretion, as an utterance boundary, if it included time enough for an inhalation, even though none was heard.

The real-time coding of utterances included 9 options for categorization of each utterance: vocant, squeal, growl, ingress, whisper, cry, whimper, laugh, and other (which included voiceless raspberries, voiced ones being categorized by their phonatory properties as vocant, squeal or growl). Ingress, whisper, laugh, and other constituted only ~1% of the coded utterances, and were not included in the analyses for the present paper. Of note, surprisingly low rates of laughter have also been reported in prior work from our group, even in cases of laboratory recordings where parents play with their infants [2, 3]. Cries, whimpers, and laughs were also comparatively infrequent (constituting <14%) of utterances, with the three primary protophones (vocants, squeals, and growls) accounting for approximately ~85% of all utterances across the first year. The criteria for categorization of cry and whimper utterances is explained in detail in a prior paper, along with acoustic specifications [4].

The present study focused on clustering of squeals and growls with regard to the very frequently occurring vocants, which constituted 74% of all utterances in the sample according to the coders, and more than 80% of the 3 phonatory protophone types. Growls in the present work, with its real-time coding, turned out to be about 7% of all utterances and squeals about 5%. This rate of squealing and growling is lower than has been found in studies using repeat-observation coding of laboratory recordings, e.g., 14% squeals and 17% growls in [3]. Our preferred interpretation of this discrepancy is that real-time coding (with only one opportunity for judgment per utterance) results in a tendency of coders to choose the “default” vocant category even more frequently than they do when listening to each utterance more than once. Thus, we presume that real-time coding tends to restrict the number of judgments of the non-default phonatory categories (squeal and growl) to particularly salient instances of their occurrence. We also think this restriction may result in a kind of recognition of clustering that may resemble the recognition by parents, who like the real-time coders, have only one opportunity with each infant utterance to gauge whether it represents a salient category.

Instructions to coders discouraged the inclusion in the coding of sounds that were so quiet or short in duration that they would be likely not noticed by caregivers. The basic approach to coding, includes the assumption that coders (almost all of whom are female in our studies) can and should listen and judge baby sounds the way caregivers do. After all, parents provide important influences on the infant in language adaptation, and if they could not make good judgments about the significance of infant vocalizations, they would surely be at a disadvantage in guiding their infants’ development. As a consequence, they should be at a disadvantage in promoting their own genes into subsequent generations, since their infants would presumably be at a disadvantage in survival and reproduction.

In the following sections, we provide definitions for vocants, squeals and growls. Buder et al. [5] and Oller [6] provide additional information regarding definitions of infant vocalizations (and particularly regarding the phonatory protophones) used in our laboratories. It is important to offer some explanation about the coding of the utterances because they are complex, yielding much better than chance agreement among coders, but also plenty of disagreement. There is considerable coding ambiguity, given that the categories are not fully discrete, but instead represent fuzzy classes with gradations among them, and often include within-utterance changes from one regime of phonation to another, yielding the basis for many coder disagreements. We reason that these disagreements are to be expected because the categories emerge, not from fixed innate inclinations, but from infant exploration within a complex space of vocal capabilities and tendencies.

All the figures presented in the Supporting Information were extracted from LENA recordings, and the spectrograms in the bottom panels of each figure are displayed at an 8 kHz range with a 30 Hz analysis bandwidth.

Vocants: The term “vocants” [7] in the present study includes both “quasivowels” and “full vowels” (also called “fully-resonant nuclei”, [6]). Vocants are presumed precursors to mature vowels in natural languages. Quasivowels are produced with a vocal tract at rest, while full vowels are produced with a postured vocal tract [6]. Both quasivowels and full vowels are produced with normal phonation and fall within the infant’s habitual pitch range [5] which is typically 350-500 Hz [8]. The vocal “regime” of vocants is also often referred to as “modal” [5].

S1 Fig a and b provide oscillographic (time domain, top half of each panel) and spectrographic (spectral domain, bottom half of each panel) portrayals of two utterances judged as vocants from LENA recordings of 3-month-old infants. All the spectrographic displays in the

figures in Supporting Information were produced from the original LENA recordings and display the full 8 kHz range available with a spectrographic bandwidth of 30 Hz, which allows for visualization of harmonics of the infant voices. As illustrated, the most prototypical vocants have clear harmonic structure with relatively little interharmonic noise. The utterances in the figure are both over a second in duration, and both are interpreted by coders as possessing at least two syllable prominences, but many vocants are much shorter, often 100 ms or less, and are often perceived as having only one syllable.

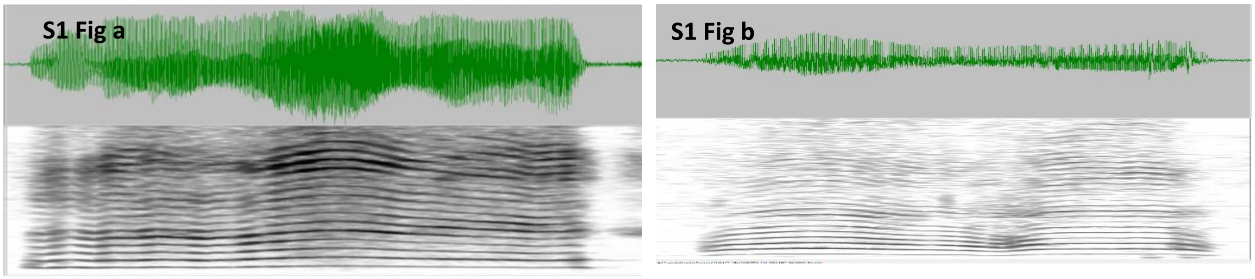

S1 Fig. Display of vocant examples: a. A vocant from a 3-month-old infant, with a duration of display of 1269 ms. Notice the harmonics are clearly visible across the entire range of the utterance, with relatively little interharmonic noise. **b.** Another vocant from a different 3-month-old, with a duration of display of 1249 ms, again showing clear harmonics and little interharmonic noise.

Growls: Growls tend to have lower pitch, sometimes much lower pitch than vocants. An utterance with pitch substantially higher than typically accompanies the modal regime in any infant is not allowed to be categorized as a growl within our scheme. The vocal regimes of growls often yield an impression of substantial harshness as corresponds to the majority of the utterance represented in S2 Fig a. An acoustic manifestation of the harshness is the considerable amount of interharmonic noise, which can be the product of chaotic, subharmonic or biphonation regimes [5]. S2 Fig b presents another way that growls can manifest, namely with extremely low pitch, where individual pitch periods can be discerned in the time domain display, and where the ear perceives a “zipper” quality attributable the listener’s being able to hear the individual pitch

pulses. This regime is thus often called "pulse" although linguists tend to term it "vocal fry" or "creaky voice."

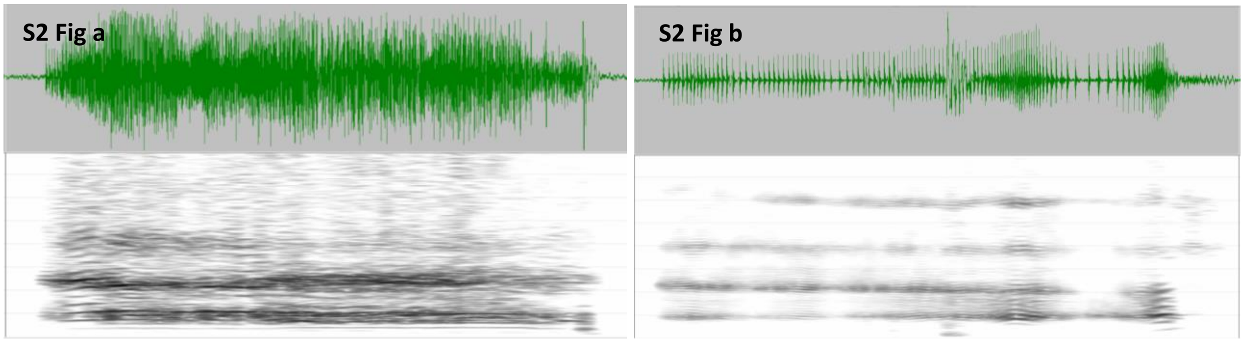

S2 Fig. Display of growl examples: a. A harsh growl from a 3-month-old infant, with a duration of display of 1215 ms. During this utterance there is considerable interharmonic noise, which contributes to the perception of phonatory harshness. The first fifth of the utterance does not show the harshness of the rest of the utterance. **b.** A second growl from a different 3-month-old, with a duration of display of 1271 ms, is very different from the one in panel a, because this one has the phonatory property of pulse (or vocal fry) throughout. Notice that harmonics in 2b are very narrowly spaced and that individual pitch periods can easily be discerned in the time domain display at the top. In both 2a and 2b coders tend to hear the pitch as being considerably lower than in vocants.

Squeals: S3 Fig a and b show squeals from two 3-month-old infants. The vocal quality in S3 Fig a corresponds to falsetto (or "loft") phonation, as indicated by very widely spaced harmonics compared with the vocants of 1a and 1b. S3 Fig b is referred to in our laboratory as a harsh squeal, which has periods of relatively pure falsetto with little noise between harmonics, but the most salient parts of the utterance are very harsh and high in pitch. There are even periods of normal phonation during this long and complex utterance. The training in our laboratories encourages coders in the forced choice real-time coding to categorize utterances with such complexities in accord with their most salient features. In the case of 3b, coders tend strongly to code the utterance as a squeal.

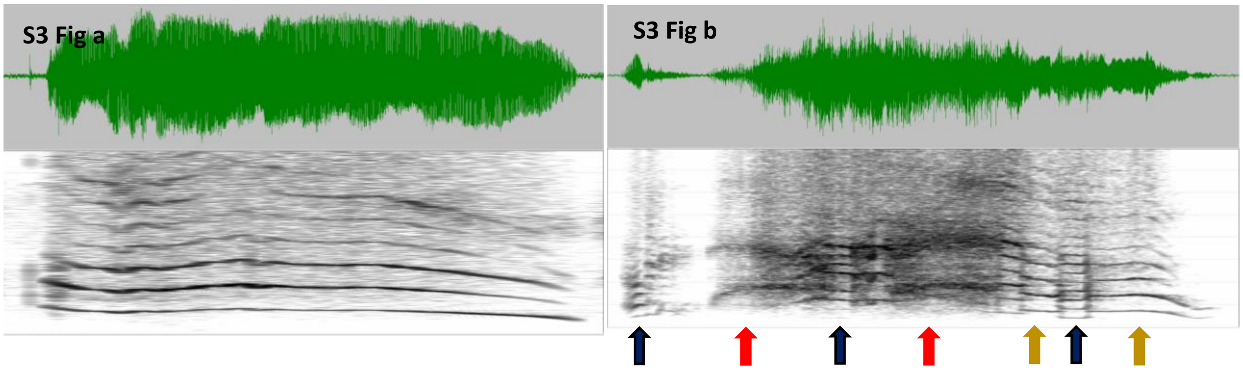

S3 Fig. Display of squeal examples: a. A squeal from a 3-month-old infant, with a duration of display of 1270 ms. Notice the harmonics that are widely spaced, corresponding to very high pitch accompanying the falsetto or "loft" regime and like the prior vocant examples, showing relatively little interharmonic noise. **b.** A much more complicated squeal from a different 3-month-old, with a duration of display of 3141 ms, showing clear widely spaced harmonics with relatively little noise between harmonics only during two periods designated by gold arrows. The red arrows indicate periods of noisy, harsh squeal phonation, with a great deal of noise, perceived when isolated as very high in pitch. Two brief periods of relatively modal phonation, with more narrowly spaced harmonics are designated by black arrows, and if isolated, sound like vocants. There is a brief period shortly after the initial period of normal phonation where there is an apparent glottal hold, resulting in very little sound. The utterance as a whole was unambiguously categorized by coders as a squeal, because the most salient auditory features of the utterance were deemed to include very high pitch. We encouraged coders to consider any salient period of squeal or growl phonation during an utterance as reason to avoid the vocant category, although there are plenty of cases where low intensity or short squeal or growl phonatory patterns are found in an utterance that coders judge as vocant.

Utterances with notable regime shifts yielding ambiguity of judgments: The coding task required a forced choice at the utterance level, and consequently coders were not uncommonly faced with utterances that had multiple possible interpretations, and sometimes, unlike the utterance in S3 Fig b (where the tendency was strong to choose squeal even given the regime complexity), there was more basis for coder disagreement. S4 Fig a and b provide good examples of hard-to-judge utterances. In both 4a and 4b we see multiple regime shifts. Coders are variable in their decisions about both these utterances, sometimes choosing squeal and sometimes growl, but never vocant.

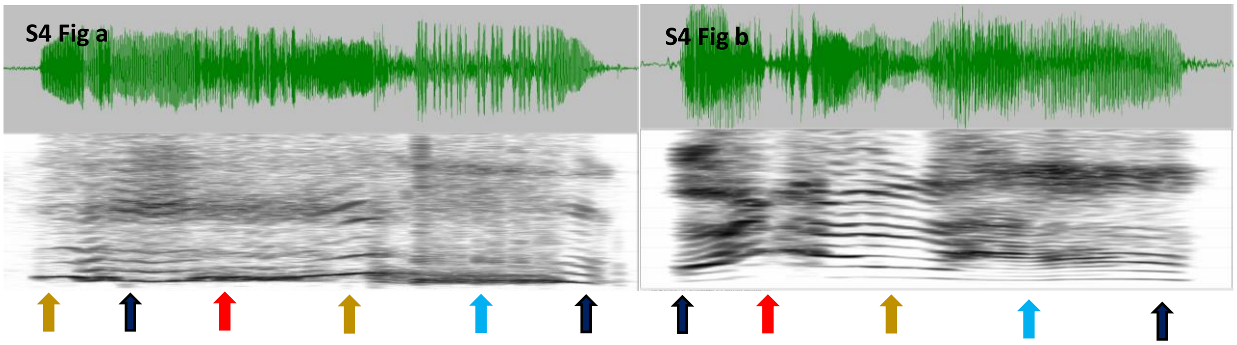

S4 Fig. Display of complex utterances with uncertain interpretation: a. A complex utterance from a 6-month-old infant, with a duration of display of 1384 ms. As before, gold arrows point to periods of loft phonation, red to periods of high-pitched harsh phonation, and black to periods of normal phonation. Blue arrows correspond to harsh growl phonation. This is an utterance that is variably coded as squeal or growl. **b.** Again, a complex utterance, 863 ms duration of display, from a different 6-month-old infant, shows several regime shifts and is judged variably as squeal or growl. These utterances would never be judged as vocants in the OLL.

Spectrographic illustrations of clustering: In S5 Fig a and b we present examples of the clustering phenomenon. In S5 Fig a, 7 squeals occur in a row, in S5 Fig b, 6 vocants in a row. In these cases, the utterances occur in a repetitive pattern for squeals in one case and vocants in the other. In the present study, clustering did not require repetition, because we counted the number of utterances of each phonatory type within 5-minute segments of recording, and then compared 5-minute segments for the numbers of each phonatory type across the segments drawn from each all-day recording. Repetition could result in significant clustering in our analysis, but clustering could also occur when the rate of squeals or growls was higher in some segments than in others in spite of the occurrence of mixing with other vocal types within the segment.

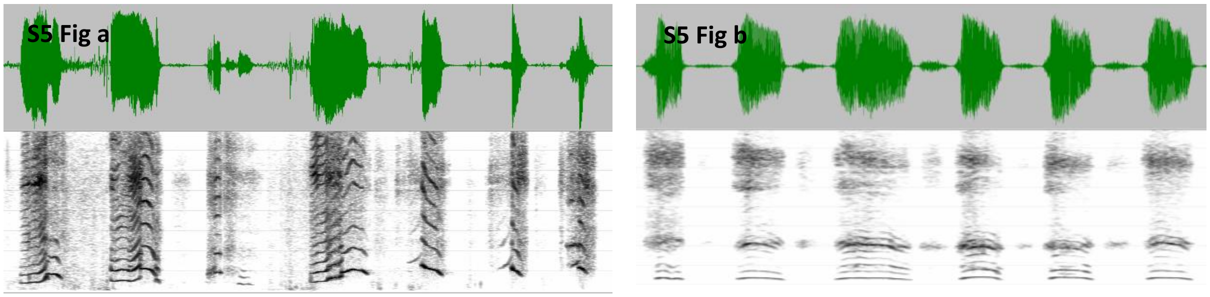

S5 Fig. Clustering Acoustic Display: a. A string of 7 utterances, all judged as squeals, from a 6-month-old infant. Notice the brief pauses between utterances, and notice that each utterance has a notable period of high pitch (falsetto), which forces the squeal judgment in accord with our coding criteria, in spite of the fact that modal phonation also occurs in several of the utterances. The duration of display is 6308 ms. **b.** From the same infant in panel a, we see a sequence of 6 vocants from the same recording day, all with clear modal phonation, and in this case there are visible inhalations between the utterances in most cases.

Illustrations of the application of Fisher's exact test

S1 Tables a and b present examples from the real data of application of the Fisher's exact test for two different recordings from two of the infants where the comparison is between squeal and vocant counts. Note that in S1a there are 14 surviving segments out of the 21 selected from each recording (segments having at least one protophone of each of the relevant types, segments not having more than 5 cries or whimpers, and segments where the infant was not asleep), while in S1b there are 12. In both cases the Fisher's exact test yielded a significant result ($p < .001$), indicating that squeals and vocants did not distribute randomly with respect to each other. Notice for the example in S1 Table a that in segments 1, 4, 19 and 20, vocants dominated, while in 16 through 18 squeals dominated, suggesting a period of squeal practice/vocal play during segments 16 and 18. Similarly in S1 Table b, the infant shows clustering of squeals in segments 18 through 20. S1 Table c shows no statistically significant pattern of clustering of squeals.

S1 Table. Examples of significant (S1a, S1b) and non-significant (S1c) clustering patterns of squeals

S1 Table a: Infant 1

Segment #	Squeal	Vocant
1. S01	17	108
2. S03	1	4
3. S04	8	80
4. S06	0	4
5. S12	5	7
6. S13	0	8
7. S14	1	5
8. S15	0	2
9. S16	23	4
10. S17	29	6
11. S18	13	6
12. S19	5	131
13. S20	3	99
14. S21	0	0

S1 Table b: Infant 2

Segment #	Squeal	Vocant
1. S01	0	66
2. S02	1	14
3. S03	1	12
4. S08	1	24
5. S09	0	4
6. S10	0	16
7. S11	1	47
8. S12	0	2
9. S18	8	49
10. S19	28	72
11. S20	6	70
12. S21	1	24

S1 Table c: Infant 3

Segment #	Squeal	Vocant
1. S01	0	1
2. S02	0	1
3. S03	0	1
4. S04	4	1
5. S05	4	1
6. S06	8	3
7. S10	0	1
8. S11	0	1
9. S12	0	1
10. S13	0	1
11. S14	0	1
12. S15	0	1
13. S16	0	1
14. S17	0	1

S1 Table: a. Numbers of squeals coded in 5-minute recording segments surviving the analysis filter (which excluded sleep segments and segments with 5 or more cries or whimpers) are contrasted with numbers of vocants in the same segments for one infant's all-day recording. Note that some segments are missing in the tables, because they did not survive the filtering. The numbering reflects the sequential order of the surviving segments. It is clear that squealing tended to occur non-randomly with respect to vocants. **b.** In a recording of an additional infant, the sharp tendency for squeals to occur non-randomly with respect to vocants across surviving segments is also obvious. **c**. In the case of another infant's recording, the Fisher's exact test did not yield a significant result (p = .264).

S2 Tables a and b present an example of application of the Fisher's exact test where the comparison is between growl and vocant counts. In S2 Table a, there were 19 surviving segments, where segments 5, 14 and 21 showed significant growl activity with respect to vocant activity, but not in any of the other segments. Similarly, in S2 Table b, where there were 18 surviving segments, there was high growl activity with respect to vocant activity in segments 8 and 21, for example. Again, for both infant 4 and infant 5, the Fisher's exact test yielded a significant result ($p < .001$), indicating that growls and vocants did not distribute randomly with respect to each other. S2 Table c shows no statistically significant pattern of growl clustering.

S2 Table. Examples of significant (S1a, S1b) and non-significant (S1c) clustering patterns of growls.

S2 Table a: Infant 4

Segment #	Growl	Vocant
1. S01	8	23
2. S02	7	51
3. S03	1	23
4. S04	3	22
5. S05	19	44
6. S06	3	49
7. S07	4	40
8. S08	0	16
9. S10	3	26
10. S11	4	28
11. S12	5	50
12. S13	5	38
13. S14	16	44
14. S15	3	53
15. S17	8	23
16. S18	1	8
17. S19	1	8
18. S20	1	46
19. S21	15	47

S2 Table b: Infant 5

Segment #	Growl	Vocant
1. S01	0	2
2. S02	3	5
3. S03	4	16
4. S04	0	0
5. S05	3	34
6. S07	0	6
7. S08	20	23
8. S10	5	26
9. S11	0	27
10. S12	4	12
11. S13	1	7
12. S15	2	49
13. S16	0	8
14. S17	0	12
15. S18	3	11
16. S19	1	22
17. S20	8	64
18. S21	14	32

S2 Table c: Infant 6

Segment #	Growl	Vocant
1. S01	0	0
2. S02	0	1
3. S03	0	2
4. S04	0	1
5. S05	1	1
6. S06	0	0
7. S08	2	5
8. S09	0	9
9. S10	2	2
10. S14	0	3
11. S15	0	5
12. S16	0	0
13. S17	0	0
14. S18	0	1
15. S19	1	5
16. S20	1	2

S2 Table: **a.** Numbers of growls coded in 5-minute recording segments surviving the analysis filter are contrasted with numbers of vocants in the same segments for an all-day recording from infant 4. It is clear that growling tended to occur non-randomly with respect to vocants. **b.** In a recording of infant 5, the sharp tendency for growls to occur non-randomly with respect to vocants across surviving segments is also obvious. **c.** In a recording of infant 6, on the other hand, the result was not statistically significant, although there were some growling sounds produced across the segments (p = .337).

Coder agreement

Results from correlational analyses illustrating statistically significant coder agreement

Providing a basis for determining the reliability of the coding of the three protophone types, 21 five-minute segments randomly selected from 9 day-long recordings (a training and agreement recording set) were coded independently by many individuals during the training period. The data from this training dataset supply a basis for an extensive evaluation of coder agreement, where all the coders who contributed to the Results of the present study participated, and where the agreement recordings were from the same Marcus Autism Center database as the recordings used in the main study.

All 21 segments of some of the 9 recordings were coded independently by all of the 36 coders of the teams that coded the data reported in Results. There were additional coders from related OLL projects who had been trained the same way who also coded some or all of the 9 recordings. Thus, there was a variable number of individuals who coded each of the 9 recordings, with an average of 32.1 coders per recording (range 20-48). These agreement recordings were essentially identical to those of the data from the 312 infants described in Methods of the main text. The recordings were also obtained with the LENA system during the same time period of the recordings from the 312 infants of the broader set evaluated by the OLL, from infants

recruited at the Marcus Autism Center in the same way, and meeting the same inclusionary and exclusionary requirements.

An additional agreement analysis is made possible by a dataset involving 9 of the coding team members who contributed to the Results. Each of these coding team members was assigned, after each one of them completed coding all the recordings on at least 4 infants to independently code semi-randomly selected 5-minute segments that had previously been coded by one of the other team members. This second dataset provided another opportunity to demonstrate that coders agreed substantially on the categorization of squeals, growls, and vocants.

But before considering the statistically significant agreement results, it is important to acknowledge that there was considerable variation among the coders in their counts of the three protophone types within recordings. This variation indicated, as was clear during the training and in prior research with such data [3], that coders often disagreed about categorizations of squeals, growls and vocants. A simple measure of the extent of this disagreement can be obtained from the coefficient of variation (CoV, ratio of standard deviation to mean) across coders for each of the 9 recordings where multiple individuals coded the same 21 segments independently. The mean CoV for vocants was 0.21 across the 9 recordings, suggesting a relatively narrow range of vocant counts across coders. Squeals and growls showed a much larger range, with mean CoVs of 0.59 and 0.96 respectively. Thus, the data for the present study are clearly subject to substantial differences among coders in how they made decisions, with the acoustically analyzed examples presented above hopefully supplying perspective on why we assert that such disagreements are to be expected. The higher CoV for growls is an indication that coder agreement was lower for growls than for squeals.

The analysis conducted on clustering and presented in Results can be justified by its much higher than chance level agreement among coders on the segment-by-segment counts of the protophone categories. We evaluated correlations among coders on squeals, segment-by-segment within recordings, as well as correlations among coders on growls. Spearman rank order correlations were computed on the 21 segments for each recording in this agreement set, first on the numbers of squeals and then on the numbers of growls classified by each coder in all the available pairwise comparisons of coders for each recording from the agreement set. In this way we determined the extent to which coding from each individual matched that of each other individual coder with regard to numbers of squeals or growls, 5-minute segment by 5-minute segment, across all 21 segments for each recording (there were 9802 individual correlations). Then we conducted that same analysis on the 5-minute segments of the second agreement set.

The analysis on the first set yielded many statistically significant correlations (for each pairwise comparison, N = 21, and for $\rho > .433$, $p < .05$). An average of 88% of pairwise comparisons produced statistically significant correlations for the 9 recordings on squeals; for growls, 45% of pairwise comparisons were statistically significant. The mean Spearman correlation across the 9 recordings for squeals was $\rho = .71$ and for growls $\rho = .42$.

To demonstrate that this degree of agreement among coders is very much greater than chance, we bootstrapped randomizations of the observed counts for all the coders for all the segments within each recording, using two of the 9 recordings for the randomization tests. The randomizations produced much smaller percentages of significant correlations for the bootstrap coders than the real coders, averaging only 6% of significant pairwise comparisons for the bootstraps on both squeals and growls, with upper bounds of their 95% confidence intervals at 7%. The lowest percentage of significant correlations between real coders for the pairwise

comparisons for any of the 9 recordings was 40% for squeals and 27% for growls, far higher than the upper bounds of the significant correlations for the bootstrapped randomization tests.

In addition, data from the second agreement study were analyzed. The agreement data were obtained near the end of the data collection from 40 of the infants who were coded by the Memphis team. Among the 36 trained coders, 10 individuals coded the 40 infants, and 9 of those coders were available to be assigned for agreement coding. We semi-randomly selected 523 five-minute segments from the 21 coded for each of the recordings and assigned each of these segments for blind recoding by an agreement coder, not the individual who had originally coded the segment. We balanced the assignments, to the extent possible, such that agreement coders were assigned segments pertaining to as many of the age categories and infants as possible given the coding time available for each individual—we succeeded in assigning each coder to segments from at least 5 of the 6 ages (mean = 5.67) and at least 19 of the 40 infants (mean = 20.67). The number of segments recoded by the agreement coders ranged from 19 to 119. Data on agreement for protophones, cries and laughs from this second agreement set were presented in a prior paper [2]. The average correlations for squeals and growls are presented here for the first time. The agreement levels between original and agreement coders were higher than in the case of the correlations among coders of the 9 training recordings. The mean across the nine coders on the second agreement set for squeals was $\rho = .79$ and for growls, $\rho = .65$). All but one of the individual 18 correlations was statistically significant. The higher correlations on the second agreement set appear to support our assumption that coding agreement improved with experience.

The fact that the inter-coder correlations significantly exceeded chance proves that the coders detected a reliable "signal" for squeals and growls in the data. That the correlations were

not high by traditional standards does not invalidate the comparisons presented in Results. Low coder agreement, as long as it is statistically significant, has an effect on group comparisons (in this case, across-session comparisons to determine possible clustering) that limits the possibility of detecting the sought after effect because the low coder agreement acts as noise and can produce Type II error. But if significant effects (in this case, clustering effects) are detected in spite of the noise, there is no reason to doubt the outcomes—the effects can be said to have been robust enough to have significantly exceeded the noise.

Results from permutation tests supporting intercoder agreement

Figures in this section present results from permutation tests [9] using all of the nine agreement recordings from the training period, aggregated to test for coder variation. We resampled subsets of the (at most) 48 coders' clustering results (from one to 48) without replacement 5,000 times and calculated the proportion of all nine recordings that showed significant clustering patterns for squeals and growls (panels A and B in S6 Fig) with respect to vocants to determine the empirical distribution of the significant clustering that occurred according to the Fisher's test under the null hypothesis that coder identity had no effect on the clustering outcomes. For C, the clustering patterns for either squeals or growls were taken into account, yielding the highest proportion of clustering for the three panels.

S6 Fig. **Permutation results for all agreement recordings.**

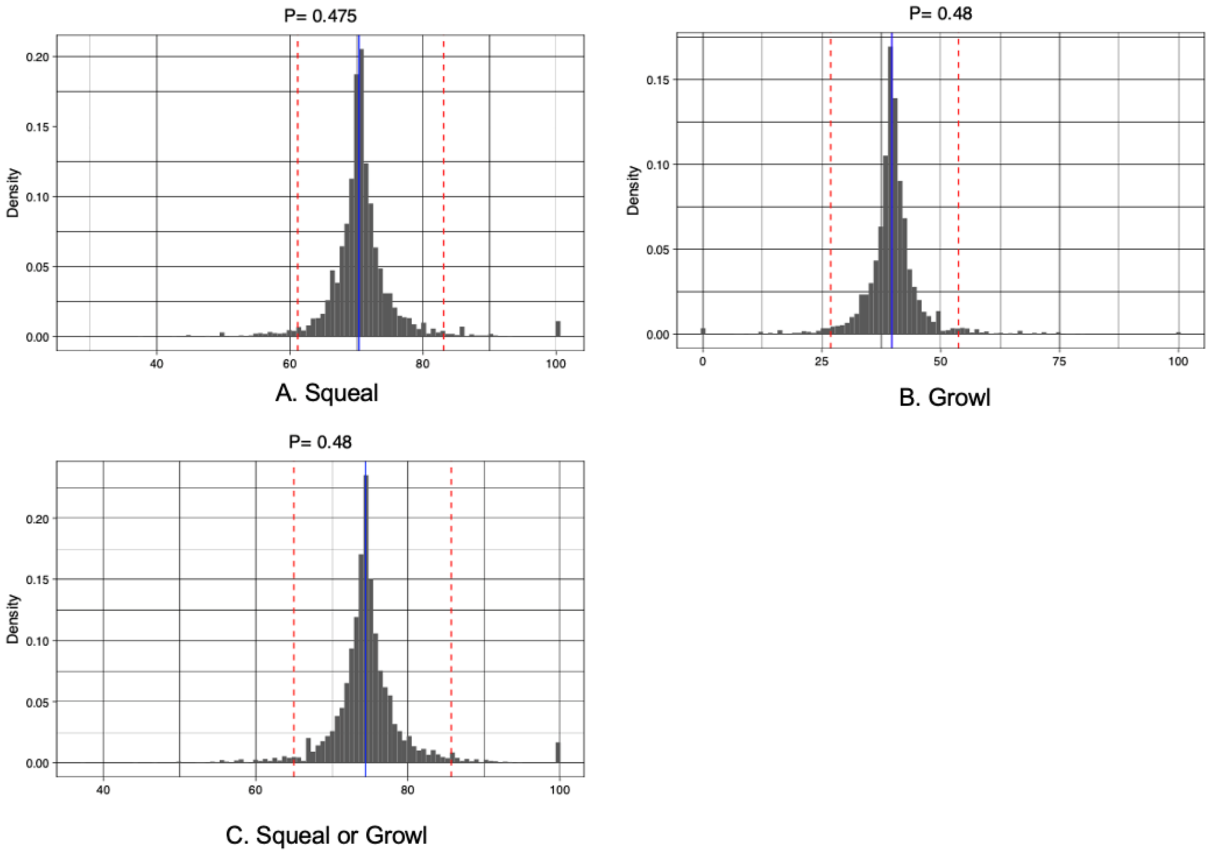

For the three panels of S6 Fig, the X-axis represents the proportion of recordings that showed significant clustering on the nine recordings. The y-axis represents the probability density for each proportion of the nine recordings that were found to have significant clustering. The blue line represents the observed mean of the actual data (with no permutation) from all the coders across all nine recordings. The red dotted lines represent the 95% confidence intervals for the distribution of proportions of recordings that showed significant clustering resulting from the permutation test. The fact that all the blue lines fall deeply within the area (and approximately on the mode) between the red dotted lines confirms that differences between coders, although present (as indicated by coefficients of variation across coders across the 9 recordings), had little effect on the significant clustering findings for these recordings. If there had been little or no

agreement among the coders, the blue lines would have fallen outside the 95% confidence interval as indicated by the dotted red lines. Thus, these findings supplied no evidence that coder differences significantly skewed the clustering results of the agreement recordings.

In separate analyses, the nine recordings were evaluated individually to produce the same kinds of permutation tests for squeals and for growls as well as for squeals or growls as shown in S6 Fig, although the number of individuals who had coded each of the recordings ranged from 20 to 48. Thus each permutation test for an individual recording was conducted just for the number of individuals who had coded all 21 segments from that recording. These analyses revealed very distinct distributions for different recordings, and even within recordings, often very different distributions for squeals and growls. For example, for recording 2 (S7 Fig) the distribution of coders' proportions of significant clustering findings for growls (a very low proportion) scarcely overlapped with the distribution of coders' proportions of significant clustering findings for squeals (a high proportion), and the confidence intervals were widely separated. In recording 9 (S8 Fig) the pattern showed very few significant findings of clustering for either squeals or growls. In recording 1 (S9 Fig) the distributions showed significant clustering for both squeals and growls, although the clustering pattern was found by the coders to be stronger for squeals. In recording 6 (S10 Fig) there was total coder agreement that squeals were clustered (and that is why the panels for squeals and squeals or growls are blanked out), while the distribution for coder findings on growls centered around 50% significant clustering. Across the nine recordings, the distributions strongly indicated that, as a group, the coders agreed on whether either squeals or growls or both showed clustering. As in the case of the aggregated analysis depicted in S6 Fig, if there had been little or no agreement among the coders, the blue lines for S7-S10 Figs could indeed have fallen outside the 95% confidence interval as indicated

by the dotted red lines. But that did not happen in any case across the nine recordings, because the coder agreement was too high.

The conclusion of these agreement analyses is that, while there was considerable inter-coder variation, the overall agreement on coding was strong and gives reason for confidence in the clustering results reported in the main text.

S7 Fig. Permutation results for Recording 2.

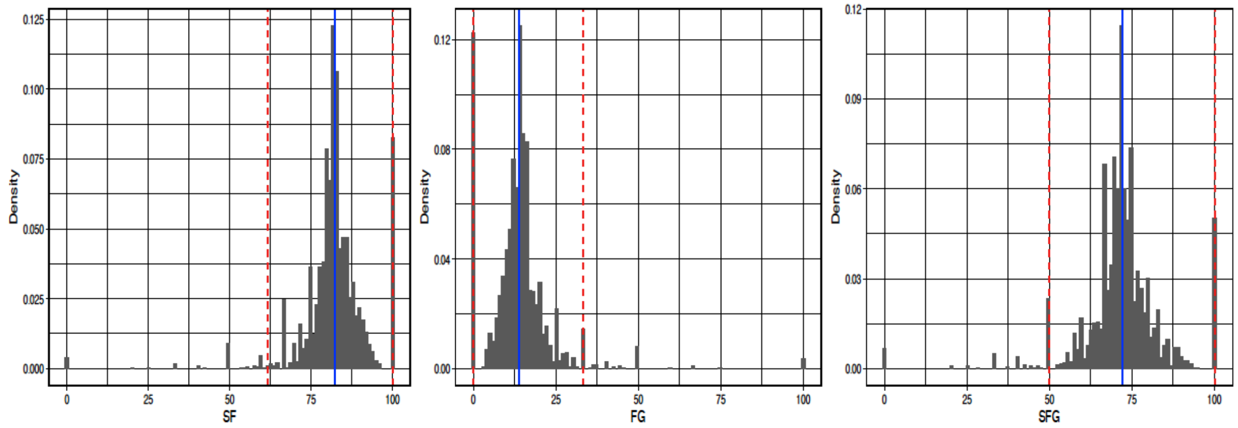

Note. The x-axis represents the percentage of recordings that showed (a) significant clustering of squeals with regard to vocants (SF); (b) significant clustering of growls with regard to vocants (FG); and (c) significant clustering of either squeals or growls with regard to vocants (SFG).

S8 Fig. Permutation results for Recording 9.

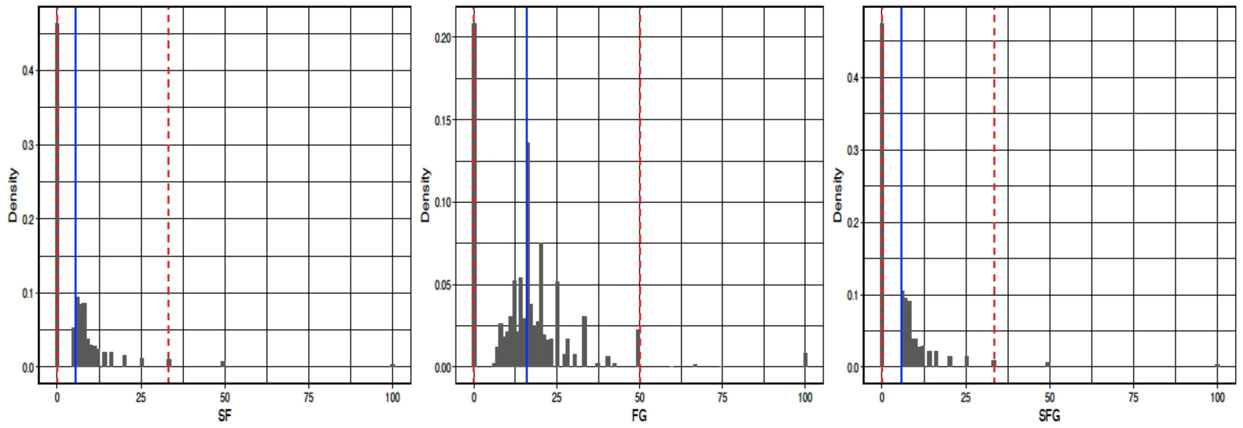

S9 Fig. Permutation results for Recording 1.

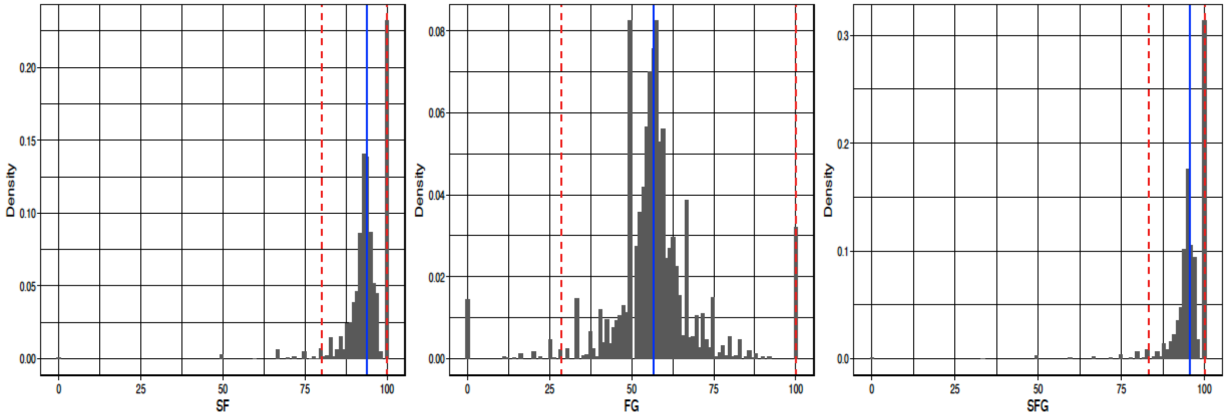

S10 Figure. Permutation results for Recording 6.

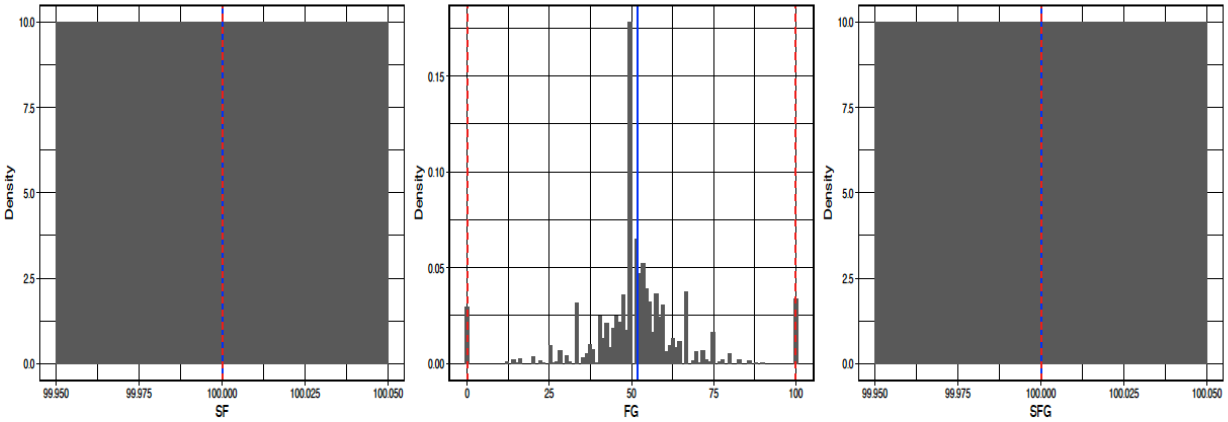

Note. The large gray area centered around 100% corresponds to perfect coder agreement. All coders showed significant clustering for squeals and consequently also for squeals or growls.

References

1. Lynch MP, Oller DK, Steffens ML, Buder EH. Phrasing in prelinguistic vocalizations. Developmental Psychobiology. 1995;28:3-23.
2. Oller DK, Ramsay G, Long HL, Griebel U. Protophones, the precursors to speech, dominate the human infant vocal landscape. Philosophical Transactions of the Royal Society B: Biological Sciences. 2021;376(1836):20200255.
3. Oller DK, Buder EH, Ramsdell HL, Warlaumont AS, Chorna L, Bakeman R. Functional flexibility of infant vocalization and the emergence of language. Proceedings of the National Academy of Sciences. 2013;110(16):6318-632.
4. Yoo H, Buder EH, Bowman DD, Bidelman GM, Oller DK. Acoustic correlates and adult perceptions of distress in infant speech-like vocalizations and cries. Frontiers in Psychology. 2019;10(1154).
5. Buder EH, Warlaumont AS, Oller DK. An acoustic phonetic catalog of prespeech vocalizations. In: MacLeod BPA, editor. Comprehensive perspectives on child speech development and disorders: Pathways from linguistic theory to clinical practice. Hauppage, NY: Nova Science Publishers, Inc. ; 2013. p. 103-34.
6. Oller DK. The Emergence of the Speech Capacity. Mahwah, NJ: Lawrence Erlbaum Associates; 2000. 428 p.
7. Martin GB, Clark RD. Distress crying in neonates: Species and peer specificity. Developmental Psychology. 1982;18:3-9.
8. Kent RD, Murray AD. Acoustic features of infant vocalic utterances at 3, 6, and 9 months. Journal of the Acoustical Society of America. 1982;72:353-65.
9. Good PI. Permutation, Parametric, and Bootstrap Tests of Hypotheses: Springer Verlag; 2005.
