# Supplementary material for: Infant vocal category exploration as a foundation for speech development
Source: PLoS One. 2024 May 29;19(5):e0299140. doi: 10.1371/journal.pone.0299140 (PMC11135693; doi:10.1371/journal.pone.0299140)
Supplement: S1 File — (ZIP) [file IRB0000059383.pdf › IRB/IRB0000059383.pdf]

TO: Gordon Ramsay, MD
Principal Investigator

DATE: November 7, 2012

RE: **Expedited Approval**
IRB00059383
The Ontogeny of Social Vocal Engagement and its Derailment in Autism

Dear Dr. Ramsay,

Thank you for submitting a new application for this protocol. This research is eligible for expedited review under 45 CFR.46.110 and/or 21 CFR 56.110 because it poses minimal risk and fits the regulatory category F[4[5]] as set forth in the Federal Register. The Emory IRB reviewed it by expedited process on 11/03/2012 and granted approval effective from **11/03/2012** through **11/02/2013**. Thereafter, continuation of human subjects research activities requires the submission of a renewal application, which must be reviewed and approved by the IRB prior to the expiration date noted above. Please note carefully the following items with respect to this approval:

- Protocol document (Version: 10/01/2012)
- Questionnaries and Surveys:
    - Autism diagnostic observation schedule- Module 1
    - Autism diagnostic observation schedule- Module 2
    - BSRC Intervention History form
    - CHARGE Family Characteristics Questionnaire
    - CHARGE Physical Examination
    - Child Behavior Checklist
    - Communication and Symbolic Behavior Scales Development Profile
    - Communication and Symbolic Behavior Scales Development Profile
    - Early Screening for Autism and Communication Disorders
    - Family Demographic Information form
    - Goldman-Fristole test of articulation
    - Infant/toddler sensory profile
    - Marcus Autism Center Maternal History form
    - Modified Checklist for Autism in Toddlers
    - Mullen scales of early learning
    - NDAR medical history questionnaire
    - NIMH Physical Exam and Neurologic Evaluation
    - Parent/Caregiver questionnaire (12 months)
    - Parent/Caregiver Questionnaire (24 months)

    - Parent/Caregiver Questionnaire (36 months)
    - Social Communication questionnaire (Current)
    - Social Communication Questionnaire (Lifetime)
    - Social Responsiveness Scale
    - Systematic Observation of red Flags of ASD
    - Vineland Adaptive Behavior Scales, Second edition
    - Contact information form
- Recruitment materials:
    - Recruitment letter
    - Recruitment flyer
    - Recruitment Brochure
- Informed Consent Forms:
    - Consent form (Mother of infant Control) (Version: 10/01/2012)
    - Consent form (Mother of infant sibling) (Version: 10/01/2012)
    - HIPAA patient Authorization form (Version: 10/01/2012)
    - Marcus Autism Center Research Participation Form (Version: 03/21/2011)
    - Permission form (Child) (Version: 10/01/2012)
    - Release of Healthcare Information Form (Adult) (Version: 10/01/2012)
    - Release of Healthcare Information for (Child) (Version: 10/01/2012)
- Revocation Letter

Any reportable events (e.g., unanticipated problems involving risk to subjects or others, noncompliance, breaches of confidentiality, HIPAA violations, protocol deviations) must be reported to the IRB according to our Policies & Procedures at www.irb.emory.edu, immediately, promptly, or periodically. Be sure to check the reporting guidance and contact us if you have questions. Terms and conditions of sponsors, if any, also apply to reporting.

Before implementing any change to this protocol (including but not limited to sample size, informed consent, study design, you must submit an amendment request and secure IRB approval.

In future correspondence about this matter, please refer to the IRB file ID, name of the Principal Investigator, and study title. Thank you

Steven J. Anzalone, M.S.
IRB Analyst Assistant
This letter has been digitally signed

CC:	Asher	Jennifer	MARCUS INSTITUTE
	Ghai	Shweta	MARCUS INSTITUTE
	Muench	Kristin	MARCUS INSTITUTE
	Jones	Warren	MARCUS INSTITUTE
	Klin	Ami	MARCUS INSTITUTE

Emory University
1599 Clifton Road, 5th Floor - Atlanta, Georgia 30322
Tel: 404.712.0720 - Fax: 404.727.1358 - Email: irb@emory.edu - Web: http://www.irb.emory.edu/
An equal opportunity, affirmative action university

EMORY UNIVERSITY | Institutional Review Board

TO: Gordon Ramsay, MD
Principal Investigator

DATE: October 10, 2013

RE: **Continuing Review Expedited Approval**
CR1_IRB00059383

IRB00059383
The Ontogeny of Social Vocal Engagement and its Derailment in Autism

Dear Dr. Ramsay,

Thank you for submitting a renewal application for this protocol. The Emory IRB reviewed it by the expedited process on 10/09/2013, per 45 CFR 46.110, the Federal Register expeditable category F[4][5], and/or 21 CFR 56.110. This reapproval is effective from **10/09/2013** through **10/08/2014**. Thereafter, continuation of human subjects research activities requires the submission of another renewal application, which must be reviewed and approved by the IRB prior to the expiration date noted above. Please note carefully the following items with respect to this reapproval:

- Informed consent- Mother of infant control (Version: 01/15/2013)
- Informed Consent- Mother of Infant sibling (Version: 01/15/2013)
- HIPAA Authorization form (Version: 01/15/2013)
- Marcus Autism center research participation form (Version: 03/21/2011)
- Permission form(Version: 01/15/2013)
- Recording permission form (Adult) (Version: 10/01/2012)
- Recording permission form (child) (Version: 10/01/2012)
- Release of healthcare information form -Adult (Version: 10/01/2012)
- Release of healthcare information form- child (Version: 10/01/2012)
- Revocation letter (Version: 10/01/2012)

Any reportable events (e.g., unanticipated problems involving risk to subjects or others, noncompliance, breaches of confidentiality, HIPAA violations, protocol deviations) must be reported to the IRB according to our Policies & Procedures at www.irb.emory.edu, immediately, promptly, or periodically. Be sure to check the reporting guidance and contact us if you have questions. Terms and conditions of

sponsors, if any, also apply to reporting.

Before implementing any change to this protocol (including but not limited to sample size, informed consent, study design, you must submit an amendment request and secure IRB approval.

In future correspondence about this matter, please refer to the IRB file ID, name of the Principal Investigator, and study title. Thank you.

Sincerely,

Steven J. Anzalone, M.S.
IRB Research Protocol Analyst
This letter has been digitally signed

CC:	Asher	Jennifer	MARCUS INSTITUTE
	Ghai	Shweta	MARCUS INSTITUTE
	Muench	Kristin	MARCUS INSTITUTE
	Jones	Warren	MARCUS INSTITUTE
	Klin	Ami	MARCUS INSTITUTE

Emory University
1599 Clifton Road, 5th Floor - Atlanta, Georgia 30322
Tel: 404.712.0720 - Fax: 404.727.1358 - Email: irb@emory.edu - Web: http://www.irb.emory.edu/
An equal opportunity, affirmative action university

TO: Gordon Ramsay, MD
Principal Investigator

DATE: October 13, 2014

RE: **Continuing Review Expedited Approval**
CR2_IRB00059383

IRB00059383
The Ontogeny of Social Vocal Engagement and its Derailment in Autism

Dear Dr. Ramsay,

Thank you for submitting a renewal application for this protocol. The Emory IRB reviewed it by the expedited process on 10/08/2014, per 45 CFR 46.110, the Federal Register expeditable category F(7)(3), and/or 21 CFR 56.110. This reapproval is effective from **10/08/2014** through **10/07/2015**. Thereafter, continuation of human subjects research activities requires the submission of another renewal application, which must be reviewed and approved by the IRB prior to the expiration date noted above. Please note carefully the following items with respect to this reapproval:

- Informed Consent form- mother of infant control (Version: 01/15/2013)
- Informed Consent form- Mother of infant sibling (Version: 01/15/2013)
- HIPAA Authorization form (Version: 01/15/2013)
- Research Participation form (Version: 03/21/2011)
- Permission form- Child (Version: 01/15/2013)
- Recording permission form- Adult (Version: 10/01/2012)
- Recording permission form - Child (Version: 10/01/2012)
- Release of healthcare information form-adult (Version: 10/01/2012)
- Release of healthcare information form- child (Version: 10/01/2012)
- Revocation letter (Version: 10/01/2012)
- Protocol document (Version: 05/15/2014)

Any reportable events (e.g., unanticipated problems involving risk to subjects or others, noncompliance, breaches of confidentiality, HIPAA violations, protocol deviations) must be reported to the IRB according to our Policies & Procedures at www.irb.emory.edu, immediately, promptly, or periodically. Be sure to check the reporting guidance and contact us if you have questions. Terms and conditions of sponsors, if any, also apply to reporting.

Before implementing any change to this protocol (including but not limited to sample size, informed consent, study design, you must submit an

amendment request and secure IRB approval.

In future correspondence about this matter, please refer to the IRB file ID, name of the Principal Investigator, and study title. Thank you.

Sincerely,

Steven J. Anzalone, M.S.
IRB Research Protocol Analyst
This letter has been digitally signed

CC:	Edwards	Aric	Marcus Center
	Ghai	Shweta	Marcus Center
	Jones	Warren	Marcus Center
	Klin	Ami	Marcus Center

TO: Gordon Ramsay, PhD
Principal Investigator
Marcus Center

DATE: October 5, 2015

RE: **Continuing Review Expedited Approval**
CR3_IRB00059383

IRB00059383
The Ontogeny of Social Vocal Engagement and its Derailment in Autism

Thank you for submitting a renewal application for this protocol. The Emory IRB reviewed it by the expedited process on **10/1/2015**, per 45 CFR 46.110, the Federal Register expeditable categories F[7] & [3], and/or 21 CFR 56.110. This re-approval is effective from **10/08/2015** through **10/07/2016**. Thereafter, continuation of human subjects research activities requires the submission of another renewal application, which must be reviewed and approved by the IRB prior to the expiration date noted above. Please note carefully the following items with respect to this reapproval:

- Please note that the following study team members have been administratively removed due to expired CITI training:

1. Jennifer Asher, Alyna Khan, Elizabeth Mitchell, Eugene Kim, and Steven Kovar

Any reportable events (e.g., unanticipated problems involving risk to subjects or others, noncompliance, breaches of confidentiality, HIPAA violations, protocol deviations) must be reported to the IRB according to our Policies & Procedures at www.irb.emory.edu, immediately, promptly, or periodically. Be sure to check the reporting guidance and contact us if you have questions. Terms and conditions of sponsors, if any, also apply to reporting.

Before implementing any change to this protocol (including but not limited to sample size, informed consent, and study design), you must submit an amendment request and secure IRB approval.

In future correspondence about this matter, please refer to the IRB file ID, name of the Principal Investigator, and study title. Thank you.

Sincerely,

Micole Hasan

IRB Analyst Assistant
This letter has been digitally signed

CC:			
	Edwards	Aric	Marcus Center
	Ghai	Shweta	Marcus Center
	Jones	Warren	Marcus Center
	Klin	Ami	Marcus Center

Emory University
1599 Clifton Road, 5th Floor - Atlanta, Georgia 30322
Tel: 404.712.0720 - Fax: 404.727.1358 - Email: irb@emory.edu - Web: http://www.irb.emory.edu/
An equal opportunity, affirmative action university

TO: Gordon Ramsay, PhD
Principal Investigator
SOM: Peds: Marcus Center

DATE: October 6th, 2016

RE: **Continuing Review Expedited Approval**
CR4_IRB00059383

IRB00059383
The Ontogeny of Social Vocal Engagement and its Derailment in Autism

Thank you for submitting a renewal application for this protocol. The Emory IRB reviewed it by the expedited process on **October 5th, 2016**, per 45 CFR 46.110, the Federal Register expeditable categories F3 and F7, and/or 21 CFR 56.110. This reapproval is effective from **October 5th, 2016** through **October 4th, 2017**. Thereafter, continuation of human subjects research activities requires the submission of another renewal application, which must be reviewed and approved by the IRB prior to the expiration date noted above. Please note carefully the following items with respect to this reapproval:

- Scientific Protocol (*Version Date: May 5th, 2014*)
- Consent Form (Mother of Infant Control) (*Version Date: July 15th, 2015*)
- Consent Form (Mother of Infant Sibling) (*Version Date: July 15th, 2015*)
- Contact Information Form (*Version Date: July 15th, 2014*)
- Marcus Autism Center Research Participation Form (*Date modified: October 2nd, 2012*)
- Payment Card Information Form (*Date modified: December 18th, 2013*)
- Permission Form (Child) (*Version Date: July 15th, 2015*)
- Recording Permission Form (Adult/Child) (*Version Date: July 15th, 2014*)
- Release of Healthcare Information Form (Adult) (*Version Date: July 15th, 2014*)
- Release of Healthcare Information Form (Child) (*Version Date: July 15th, 2014)*
- Revocation Letter (*Version Date: July 15th, 2014)*

Any reportable events (e.g., unanticipated problems involving risk to subjects or others, noncompliance, breaches of confidentiality, HIPAA

violations, protocol deviations) must be reported to the IRB according to our Policies & Procedures at www.irb.emory.edu, immediately, promptly, or periodically. Be sure to check the reporting guidance and contact us if you have questions. Terms and conditions of sponsors, if any, also apply to reporting.

Before implementing any change to this protocol (including but not limited to sample size, informed consent, and study design), you must submit an amendment request and secure IRB approval.

In future correspondence about this matter, please refer to the IRB file ID, name of the Principal Investigator, and study title. Thank you.

Sincerely,

Parul Reddy
Analyst Assistant
This letter has been digitally signed

CC:			
	Edwards	Aric	SOM: Peds: Marcus Center
	Ghai	Shweta	SOM: Peds: Marcus Center
	Jones	Warren	SOM: Peds: Marcus Center
	Klin	Ami	SOM: Peds: Marcus Center

TO: Gordon Ramsay, PhD
Principal Investigator
SOM: Peds: Marcus Center

DATE: October 4, 2017

RE: **Continuing Review Expedited Approval**
CR5_IRB00059383

IRB00059383
The Ontogeny of Social Vocal Engagement and its Derailment in Autism

Thank you for submitting a renewal application for this protocol. The Emory IRB reviewed it by the expedited process on 10/4/2017, per 45 CFR 46.110, the Federal Register expedited review categories F(4, 5, 6, and 7), and/or 21 CFR 56.110. Research involving minor subjects is approved under 45 CFR 46.404.This reapproval is effective from **10/4/2017** through **10/3/2018**. Thereafter, continuation of human subjects research activities requires the submission of another renewal application, which must be reviewed and approved by the IRB prior to the expiration date noted above.

The following consent documents are approved for use in the new approval period:

- Consent Form (Mother of Infant Control) (*Version Date: July 15th, 2015*)
- Consent Form (Mother of Infant Sibling) (*Version Date: July 15th, 2015*)
- Contact Information Form (*Version Date: July 15th, 2014*)
- Marcus Autism Center Research Participation Form (*Date modified: October 2nd, 2012*)
- Payment Card Information Form (*Date modified: December 18th, 2013*)
- Permission Form (Child) (*Version Date: July 15th, 2015*)
- Recording Permission Form (Adult/Child) (*Version Date: July 15th, 2014*)
- Release of Healthcare Information Form (Adult) (*Version Date: July 15th, 2014*)
- Release of Healthcare Information Form (Child) (*Version Date: July 15th, 2014)*
- Revocation Letter (*Version Date: July 15th, 2014)*

Any reportable events (e.g., unanticipated problems involving risk to subjects or others, noncompliance, breaches of confidentiality, HIPAA violations, protocol deviations) must be reported to the IRB according to our Policies & Procedures at www.irb.emory.edu, immediately, promptly, or periodically. Be sure to check the reporting guidance and contact us if you

have questions. Terms and conditions of sponsors, if any, also apply to reporting.

Before implementing any change to this protocol (including but not limited to sample size, informed consent, and study design), you must submit an amendment request and secure IRB approval.

In future correspondence about this matter, please refer to the IRB file ID, name of the Principal Investigator, and study title. Thank you.

Sincerely,

Sam Roberts, BA
Research Protocol Analyst, Sr.
This letter has been digitally signed

CC:			
	Edwards	Aric	SOM: Peds: Marcus Center
	Edwards	Morgan	SOM: Peds: Marcus Center
	Ghai	Shweta	SOM: Peds: Marcus Center
	Jones	Warren	SOM: Peds: Marcus Center
	Klin	Ami	SOM: Peds: Marcus Center

TO: Gordon Ramsay, PhD
Principal Investigator
SOM: Peds: Marcus Center

DATE: October 9, 2018

RE: **Continuing Review Expedited Approval**
CR6_IRB00059383

IRB00059383
The Ontogeny of Social Vocal Engagement and its Derailment in Autism

Thank you for submitting a renewal application for this protocol. The Emory IRB reviewed it by the expedited process on 10/9/2018, per 45 CFR 46.110, the Federal Register expedited review categories F(4, 5, 6, and 7), and/or 21 CFR 56.110. This reapproval is effective from **10/9/2018** through **10/8/2019**. Thereafter, continuation of human subjects research activities requires the submission of another renewal application, which must be reviewed and approved by the IRB prior to the expiration date noted above.

The following consent documents are approved for use in the new approval period:

- Consent/HIPAA Authorization form (Mother of Infant Control), version date 07/15/2015
- Consent/HIPAA Authorization form (Mother of Infant Sibling), version date 07/15/2015
- Consent/HIPAA Authorization form (Infant), version date 07/15/2015
- Permission form for Use of Audio/Video/Photographic Recordings: Adult & Child Participant, version date 07/15/2014
- Release of Healthcare Information form: Adult, version date 07/15/2014
- Release of Healthcare Information form: Child, version date 07/15/2014
- Revocation letter, version date 07/15/2014

Any reportable events (e.g., unanticipated problems involving risk to subjects or others, noncompliance, breaches of confidentiality, HIPAA violations, protocol deviations) must be reported to the IRB according to our Policies & Procedures at www.irb.emory.edu, immediately, promptly, or periodically. Be sure to check the reporting guidance and contact us if you have questions. Terms and conditions of sponsors, if any, also apply to reporting.

Before implementing any change to this protocol (including but not limited to sample size, informed consent, and study design), you must submit an amendment request and secure IRB approval.

In future correspondence about this matter, please refer to the IRB file ID, name of the Principal Investigator, and study title. Thank you.

Sincerely,

Sam Roberts, BA
Research Protocol Analyst, Sr.
This letter has been digitally signed

CC:	Edwards	Morgan	SOM: Peds: Marcus Center
	Ghai	Shweta	SOM: Peds: Marcus Center
	Trumbull	Ashley	SOM: Peds: Marcus Center

Jones	Warren	SOM: Peds: Marcus Center
Klin	Ami	SOM: Peds: Marcus Center

Emory University
1599 Clifton Road, 5th Floor - Atlanta, Georgia 30322
Tel: 404.712.0720 - Fax: 404.727.1358 - Email: irb@emory.edu - Web: http://www.irb.emory.edu/
An equal opportunity, affirmative action university

TO: Gordon Ramsay, PhD
Principal Investigator
SOM: Peds: Marcus Center

DATE: October 3, 2019

RE: **Continuing Review Expedited Approval**
CR7_IRB00059383

IRB00059383
The Ontogeny of Social Vocal Engagement and its Derailment in Autism

Thank you for submitting a renewal application for this protocol. The Emory IRB reviewed it by the expedited process on **10/2/2019**, per 45 CFR 46.110, the Federal Register expeditable categories F[4], F[5], F[6], F[7], and/or 21 CFR 56.110. This reapproval is effective from **10/2/2019** through **10/1/2020**. Thereafter, continuation of human subjects research activities requires the submission of another renewal application, which must be reviewed and approved by the IRB prior to the expiration date noted above. Please note carefully the following items with respect to this reapproval:

- Protocol
  - Scientific Protocol

- Consent Documents
  - Consent Form (Mother of Infant Control)
  - Consent Form (Mother of Infant Sibling)
  - Permission Form (Child)
  - Recording Permission Form (Adult/Child)
  - Release of Healthcare Information Form (Adult)
  - Release of Healthcare Information Form (Child)
  - Revocation Letter

Any reportable events (e.g., unanticipated problems involving risk to subjects or others, noncompliance, breaches of confidentiality, HIPAA violations, protocol deviations) must be reported to the IRB according to our Policies & Procedures at www.irb.emory.edu, immediately, promptly, or periodically. Be sure to check the reporting guidance and contact us if you have questions. Terms and conditions of sponsors, if any, also apply to reporting.

Before implementing any change to this protocol (including but not limited to sample size, informed consent, and study design), you must submit an amendment request and secure IRB approval.

In future correspondence about this matter, please refer to the IRB file ID, name of the Principal Investigator, and study title. Thank you.

Sincerely,

Sara Choe
Analyst Assistant
This letter has been digitally signed

CC:	Edwards	Morgan	SOM: Peds: Marcus Center
	Ghai	Shweta	SOM: Peds: Marcus Center

Jones	Warren	SOM: Peds: Marcus Center
Klin	Ami	SOM: Peds: Marcus Center

IRB CONTINUING REVIEW APPROVAL

September 21, 2020

Gordon Ramsay, Ph.D.

gjramsa@emory.edu

Title:	The Ontogeny of Social Vocal Engagement and its Derailment in Autism
Principal Investigator:	Gordon Ramsay
IRB ID:	CR001-IRB00059383
Funding:	Name: Federal Agency, Emory EPEX ID: 16349, Funding Source ID: NIMH - National Institute of Mental Health
IND, IDE or HDE:	None
Documents Reviewed:	• Consent Form (Mother of Infant Control), Category: Consent Form;<br>• Consent Form (Mother of Infant Sibling), Category: Consent Form;<br>• Permission Form (Child), Category: Consent Form;<br>• Recording Permission Form (Adult/Child), Category: Consent Form;<br>• Release of Healthcare Information Form (Adult), Category: Consent Form;<br>• Release of Healthcare Information Form (Child), Category: Consent Form;<br>• Revocation Letter, Category: Consent Form;<br>• Scientific Protocol, Category: IRB Protocol;

Dear Dr. Gordon Ramsay:

Thank you for submitting a renewal application for this protocol. The Emory IRB approved it by the expedited process on 9/17/2020, per 45 CFR 46.110, the Federal Register expeditable categories F(4), F(5), F(6), F(7), and/or 21 CFR 56.110.

This reapproval is effective from 9/17/2020 through 9/16/2021. Thereafter, continuation of human subjects research activities requires the submission of another renewal application, which must be reviewed and approved by the IRB prior to the expiration date noted above.

In conducting this protocol, you are required to follow the requirements listed in the Emory Policies and Procedures, which can be found at our IRB website.

Sincerely,

Ksenia Lyon, MPA
Analyst Assistant

Your stamped consent form is available under the "Documents" tab.

Now that your submission has been approved, please take a few moments to complete the Emory IRB Satisfaction Survey*. We will use your responses to improve our service to the Emory research community. We appreciate your feedback!*

IRB CONTINUING REVIEW APPROVAL

September 9, 2021

Gordon Ramsay, PhD
SOM: Peds: Marcus Center
gjramsa@emory.edu

Title:	The Ontogeny of Social Vocal Engagement and its Derailment in Autism
Principal Investigator:	Gordon Ramsay, PhD
IRB ID:	CR002-IRB00059383
Funding:	Name: Federal Agency, Emory EPEX ID: 16349, Funding Source ID: NIMH - National Institute of Mental Health
IND, IDE or HDE:	None
Documents Reviewed:	• Consent Form (Mother of Infant Control)<br>• Consent Form (Mother of Infant Sibling)<br>• Permission Form (Child)<br>• Recording Permission Form (Adult/Child)<br>• Release of Healthcare Information Form (Adult)<br>• Release of Healthcare Information Form (Child)<br>• Revocation Letter<br>• Scientific Protocol

Dear Dr. Gordon Ramsay:

Thank you for submitting a renewal application for this protocol. The Emory IRB approved it by the expedited process on 9/8/2021, per 45 CFR 46.110, the Federal Register expeditable categories F(4), F(5), F(6), F(7)b and/or 21 CFR 56.110.

This reapproval is effective from 9/8/2021 through 9/7/2022. Thereafter, continuation of human subjects research activities requires the submission of another renewal application, which must be reviewed and approved by the IRB prior to the expiration date noted above.

<u>Please note carefully the following items with respect to this approval:</u>

- Consent Form (Mother of Infant Control)
- Consent Form (Mother of Infant Sibling)
- Permission Form (Child)
- Recording Permission Form (Adult/Child)
- Release of Healthcare Information Form (Adult)
- Release of Healthcare Information Form (Child)

- Revocation Letter
- Scientific Protocol

In conducting this protocol, you are required to follow the requirements listed in the Emory Policies and Procedures, which can be found at our IRB website.

Sincerely,

Beth Poplaski
Emory IRB Analyst Assistant

Your stamped consent form is available under the "Documents" tab.

Now that your submission has been approved, please take a few moments to complete the Emory IRB Satisfaction Survey*. We will use your responses to improve our service to the Emory research community. We appreciate your feedback!*

IRB CONTINUING REVIEW APPROVAL

September 12, 2022

Gordon Ramsay, PhD

gjramsa@emory.edu

Title:	The Ontogeny of Social Vocal Engagement and its Derailment in Autism
Principal Investigator:	Gordon Ramsay, PhD
IRB ID:	CR003-IRB00059383
Funding:	Name: Federal Agency, Emory EPEX ID: 16349, Funding Source ID: NIMH - National Institute of Mental Health
IND, IDE or HDE:	None
Documents Reviewed:	• Consent Form (Mother of Infant Control), Category: Consent Form;<br>• Consent Form (Mother of Infant Sibling), Category: Consent Form;<br>• Permission Form (Child), Category: Consent Form;<br>• Recording Permission Form (Adult/Child), Category: Consent Form;<br>• Release of Healthcare Information Form (Adult), Category: Consent Form;<br>• Release of Healthcare Information Form (Child), Category: Consent Form;<br>• Revocation Letter, Category: Consent Form;<br>• Scientific Protocol, Category: IRB Protocol;

Dear Dr. Gordon Ramsay:

Thank you for submitting a renewal application for this protocol. The Emory IRB approved it by the expedited process on 9/12/2022, per 45 CFR 46.110, the Federal Register expeditable categories F[4, 5, 6, 7(b)], and/or 21 CFR 56.110.

This reapproval is effective from 9/12/2022 through 9/11/2023. Thereafter, continuation of human subjects research activities requires the submission of another renewal application, which must be reviewed and approved by the IRB prior to the expiration date noted above. **OR** No annual IRB review is required, as permitted under the 2018 Common Rule.

Please note carefully the following items with respect to this approval:

- Consent Form (Mother of Infant Control), Category: Consent Form;
- Consent Form (Mother of Infant Sibling), Category: Consent Form;
- Permission Form (Child), Category: Consent Form;
- Recording Permission Form (Adult/Child), Category: Consent Form;
- Release of Healthcare Information Form (Adult), Category: Consent Form;
- Release of Healthcare Information Form (Child), Category: Consent Form;
- Revocation Letter, Category: Consent Form;
- Scientific Protocol, Category: IRB Protocol;

In conducting this protocol, you are required to follow the requirements listed in the Emory Policies and Procedures, which can be found at our IRB website.

Sincerely,

Chelsea Cassara, BS
IRB Analyst Assistant

Your stamped consent form is available under the "Documents" tab.

Now that your submission has been approved, please take a few moments to complete the Emory IRB Satisfaction Survey. *We will use your responses to improve our service to the Emory research community. We appreciate your feedback!*
